# Ultrasensitive assays for detection of plasma tau and phosphorylated tau 181 in Alzheimer’s disease: a systematic review and meta-analysis

**DOI:** 10.1186/s40035-021-00234-5

**Published:** 2021-03-12

**Authors:** Xulong Ding, Shuting Zhang, Lijun Jiang, Lu Wang, Tao Li, Peng Lei

**Affiliations:** 1 Department of Neurology and State Key Laboratory of Biotherapy/Collaborative Innovation Center for Biotherapy, National Clinical Research Center for Geriatrics, West China Hospital, Sichuan University, Chengdu, 610041 China; 2 Department of Neurology, West China Hospital, Sichuan University, Chengdu, 610041 China; 3 Mental Health Center and West China Brain Research Center, West China Hospital, Sichuan University, Chengdu, 610041 China; 4 Department of Rehabilitation Medicine, West China Hospital, Sichuan University, Chengdu, 610041 China

**Keywords:** Tau, Phosphorylated tau 181, Alzheimer’s disease, Simoa, IMR, EIMAF/a-EIMAF, MSD, Plasma biomarker

## Abstract

A lack of convenient and reliable biomarkers for diagnosis and prognosis is a common challenge for neurodegenerative diseases such as Alzheimer’s disease (AD). Recent advancement in ultrasensitive protein assays has allowed the quantification of tau and phosphorylated tau proteins in peripheral plasma. Here we identified 66 eligible studies reporting quantification of plasma tau and phosphorylated tau 181 (ptau181) using four ultrasensitive methods. Meta-analysis of these studies confirmed that the AD patients had significantly higher plasma tau and ptau181 levels compared with controls, and that the plasma tau and ptau181 could predict AD with high-accuracy area under curve of the Receiver Operating Characteristic. Therefore, plasma tau and plasma ptau181 can be considered as biomarkers for AD diagnosis.

**Supplementary Information:**

The online version contains supplementary material available at 10.1186/s40035-021-00234-5.

## Background

There is no cure for Alzheimer’s disease (AD). The lack of early diagnostic biomarkers for selecting prodromal or early-stage patients is one of the roadblocks in clinical trials. The National Institute on Aging—Alzheimer’s Association (NIA-AA) has recently proposed a research framework for AD and specified the importance of amyloid-beta (Aβ), tau, and neurodegeneration [AT(N)] in the biological definition of AD [1]. Although the Aβ- or tau-positron emission tomography (PET) has been developed, it is yet to be globally available, making the NIA-AA research framework challenging to put into practice. Therefore, it is urgent to discover convenient biomarkers with early-diagnostic significance.

Tau is a microtubule-associated protein localized primarily in neurons. It is also a primary component of neurofibrillary tangles (NFTs), a pathological hallmark in AD [2]. The loss of normal functions and the gain of toxic functions of Tau have been linked with the pathogenesis of AD [2–7]. Mounting evidence has suggested that the cerebrospinal fluid (CSF) levels of tau and phosphorylated tau are linearly associated with symptom severity of AD [8–11], suggesting tau as a promising biomarker for early diagnosis and prognostic prediction. However, clinical application of CSF biomarkers has been hindered by high cost, invasiveness, and side effects of lumbar punctures, such as positional headache [12].

The detection of tau in plasma has been limited due to its low abundance until recent technical development of ultrasensitive assays. The plasma tau or phosphorylated tau levels in the healthy population and diseased patients have been assessed using different technologies such as Single-molecule Array (Simoa) [13], ImmunoMagnetic Reduction (IMR) [14], enhanced immunoassay using multi-arrayed fiber optics conjugated with rolling circle amplification (a-EIMAF) [15] and Meso Scale Discovery (MSD) [16]. However, the plasma tau or phosphorylated tau levels vary among studies, and there is no cut-off threshold between AD and normal elderly. In this systematic review and meta-analysis, we set out to determine the normal range of plasma tau and phosphorylated tau 181 (ptau181) levels in healthy populations stratified by age and sex, and investigate the cut-off thresholds of plasma tau and ptau181 between AD patients and controls.

## Methods

### Literature search

Literature search was performed in databases Cochrane Library, MEDLINE, EMBASE, PubMed, Web of Science, and Google Scholar by the date of January 21st, 2021, according to the methodology suggested by the Preferred Reporting Items for Systematic reviews and Meta-Analyses (PRISMA) guidelines [17], using the following terms: Alzheimer disease, AD, dementia, Parkinson disease, PD, traumatic brain injury, TBI, plasma tau, plasma total tau, plasma phosphorylated tau, Simoa IMR, EIMAF, a-EIMAF, and MSD. Papers published in an online-first and ahead-of-print manner were included in the analysis. The protocol of the overarching project has been published (PROSPERO registration No. CRD42020151852).

### Study selection

The analysis involved three questions. For Question 1, i.e., the normal range of plasma tau/ptau181 in healthy populations, the following criteria were applied: (1) full-text publications in English; and (2) plasma tau and/or ptau181 levels were measured by Simoa, IMR, EIMAF/a-EIMAF, or MSD. Studies were excluded if: (1) without sufficient data to allow for the extraction of plasma tau/ptau181 levels, or (2) the mean age of cohorts in the studies was < 18 years.

For Question 2, i.e., the differences of plasma tau/ptau181 between AD patients and controls, the following selection criteria were applied: (1) full-text publications in English; (2) plasma tau and/or ptau181 levels were measured by Simoa, IMR, EIMAF/a-EIMAF, or MSD; and (3) AD was diagnosed according to the 2011 core clinical NIA-AA [18] or the National Institute of Neurological and Communicative Disorders and Stroke and the Alzheimer’s Disease and Related Disorders Association [19] guidelines. Studies were excluded if: (1) studies without sufficient data to allow for the extraction of plasma tau/ptau181 levels, (2) the mean age of cohorts in the studies was < 18 years, or (3) patients employed in the studies had other cognitive disorders (e.g., mild cognitive impairment [MCI], vascular dementia, and frontotemporal dementia).

Question 3 is to calculate the diagnostic accuracy of plasma tau/ptau181 for AD. For this, we analyzed all publications selected for Question 2, if the reported plasma tau/ptau181 were used for AD diagnosis in the original publications.

### Data extraction and quality assessment

Two authors (XLD and STZ) performed the study assessment independently, and data were reported following the PRISMA statement [17]. Any discrepancy would be discussed with additional reviewers (LJJ and LW). Study quality was assessed using the modified version of the Newcastle-Ottawa Scale (NOS) [20]. A score of up to 8 points was assigned to each study based on the quality of population selection, the comparability between groups, and assessment of exposure. For studies with diagnostic tests, we assessed the quality of selected literature by checking items of the Quality Assessment of Diagnostic Accuracy Studies-2 tool (QUADAS-2) [21].

### Statistical analysis and heterogeneity exploration

For the normal range of plasma tau or ptau181, the effect sizes and 95% confidence intervals (CI) were calculated using the Random-effect model, which was used to presume that the true effect size varied among studies [21]. Subgroup analysis was performed according to age and sex ratio. For the diagnostic value of plasma tau or ptau181, the weighted mean difference (WMD) with 95% CI was calculated between AD patients and controls with a random-effects model. To evaluate the diagnostic test accuracy, we used the diagnostic accuracy studies module [22] to calculate main outcome measures, including sensitivity and specificity, and the diagnostic odds ratio (DOR). We then fitted a hierarchical summary receiver operating characteristic (HSROC) model using a bivariate regression approach to visualize the relationship between sensitivity and specificity and calculated the area under curve (AUC). Heterogeneity among studies was assessed using the *I*^2^ index. According to the Cochrane handbook, the heterogeneity was classified as low (*I*^2^ index 0%–40%), moderate (30%–60%), substantial (50%–90%), and considerable (75%–100%) [23, 24]. For heterogeneity exploration, meta-regression analysis was performed to evaluate the potential bias factors, while age, sample size, sex ratio, and region were included as four covariates. Furthermore, the results of meta-analyses were assessed by funnel plots and Egger’s test [25]. All calculations were carried out using statistical softwares provided by the Cochrane Collaboration (RevMan 5.1) and Stata14.1 (StataCorp. 2015. Stata Statistical Software: Release 14. College Station, TX: StataCorp LP).

## Results

### Study inclusions and quality assessment

**Fig. 1:**
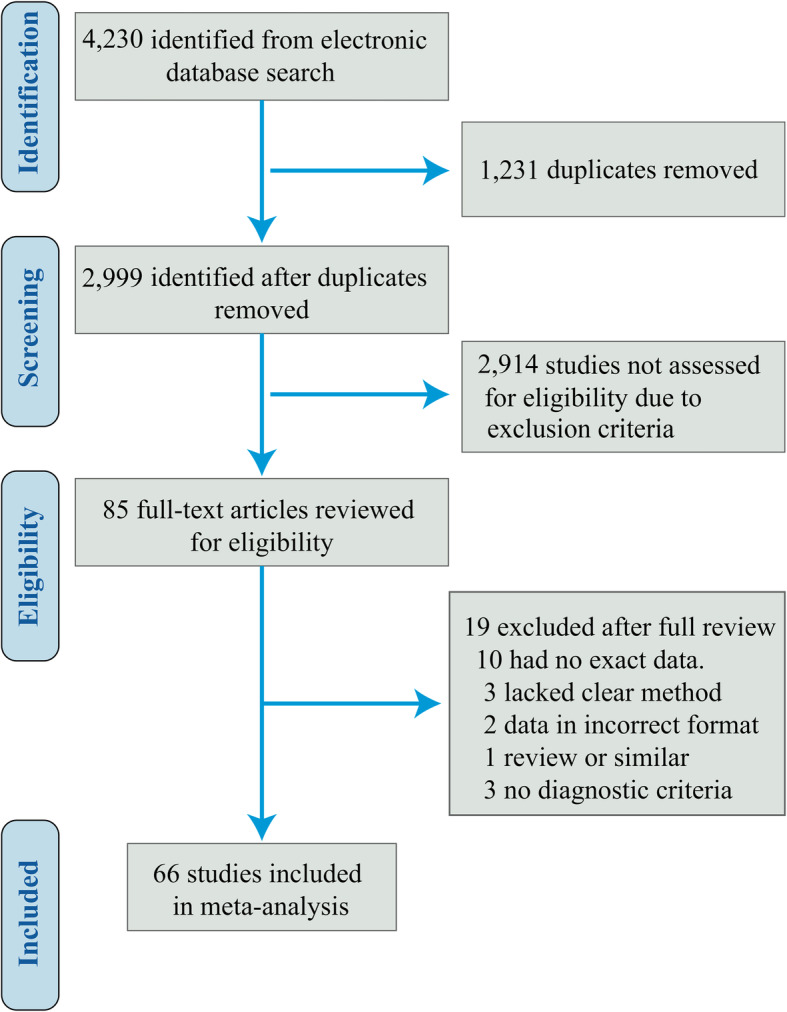
Flow chart of study selection and inclusion

The search strategy identified 4230 studies without duplication, and 2999 studies were excluded after abstract screening. Of the left 85 studies with full-text content, 19 studies were excluded as they were not a clinical study or lacked a clinical outcome description. Finally 66 studies were included in this meta-analysis (Fig. 1). Based on the modified NOS criteria [20], 53 studies were classified as high-quality, and 13 studies as medium-quality (Table S1). The revised QUADAS-2 [26] determined that the overall quality of studies included was robust. Most studies ranked as low bias and low applicability concern (Fig. S1).

The characteristics, including number of subjects, average age, male percentage, and plasma tau/ptau181 levels of the 66 studies are summarized in Tables 1 and 2. Forty-one studies using Simoa (15,490 healthy controls), 15 studies using IMR (727 healthy controls), and two studies using a-EIMAF (189 healthy controls) were included for plasma tau studies. Twenty-two studies focused on AD, comprising 1456 patients with AD and 1973 controls. Only studies using Simoa (13 studies, 1189 AD patients and 1611 controls) and IMR (9 studies, 267 AD patients and 362 controls) were included for analysis since there were insufficient data for a-EIMAF. For ptau181, six studies using the Simoa - Karikari method (not the commercial p-tau181 version) (1424 healthy controls) and three using MSD (440 healthy controls) were included for the normal range analysis. Four studies using Simoa (392 AD patients and 773 controls) and three studies using MSD (231 AD patients and 440 controls) were identified for analysis related to AD diagnosis.

**Table 1:** Basic characteristics of included studies for plasma tau analysis

Study	Subjects (***n***)	Male (%)	Age, years (mean ± SD)	Plasma tau (pg/ml)	Research question	Method
Shahim et al. (2014) [27]	CN (47)	NA	28 ± 14.07	4.5 ± 5.66	1	Simoa
Bogoslovsky et al. (2015) [28]	CN (69)	51	45 ± 15.5	4.34 ± 1.77	1	Simoa
Olivera et al. (2015) [29]	CN (28)	96.4	28.40 ± 4.47	0.63 ± 0.48	1	Simoa
Dage et al. (2016) [30]	CN (378)	61.4	80 ± 5.19	4.14 ± 1.56	1	Simoa
Alosco et al. (2017) [31]	CN (25)	100	55.16 ± 7.95	2.46 ± 0.57	1	Simoa
Mielke et al. (2017) [32]	CN (335)	62.4	80.8 ± 4.8	4.2 ± 1.5	1	Simoa
Müller et al. (2017) [33]	CN (134)	59.7	68.4 ± 5	3.6 ± 1.7	1	Simoa
Kasai et al. (2017) [34]	CN (22)	54.5	37.4 ± 12.0	0.470 ± 0.232	1	Simoa
Foiani et al. (2018) [35]	CN (22)	41	68.7 ± 6.5	1.67 ± 0.50	1	Simoa
Bergman et al. (2018) [36]	CN (36)	0	30 ± 4	6.24 ± 2.76	1	Simoa
Lippa et al. (2018) [37]	CN (42)	90.5	36.21 ± 11.69	2.81 ± 1.20	1	Simoa
Verberk et al. (2018) [38]	CN (191)	63	59 ± 9	3.18 ± 1.07	1	Simoa
Shahim et al. (2018) [39]	CN (19)	NA	25.0 ± 8.89	1.8 ± 1.48	1	Simoa
Wallace et al. (2018) [40]	CN (13)	100	18.5 ± 1.7	2.56 ± 1.02	1	Simoa
Motamedi et al. (2018) [41]	CN (24)	91.7	30.9 ± 7.77	2.48 ± 1.94	1	Simoa
Fortea et al. (2018) [42]	CN (67)	30	52.05 ± 5.50	2.23 ± 1.63	1	Simoa
Zeitlberger et al. (2018) [43]	CN (13)	46.2	37	2.08 ± 1.23	1	Simoa
Shi et al. (2019) [44]	CN (87)	41.4	64.77 ± 7.40	3.56 ± 1.84	1	Simoa
Kitaguchi et al. (2019) [45]	CN (11)	45.4	67.8 ± 3.71	0.63 ± 0.3	1	Simoa
Pase et al. (2019) [46]	CN (3232)	46.8	58 ± 14	3.93 ± 1.11	1	Simoa
Korley et al. (2019) [47]	CN (63)	63.5	39.0 ± 20.7	3.5 ± 3.19	1	Simoa
Kritikos et al. (2020) [48]	CN (398)	94.72	54.3 ± 8.1	1.67 ± 0.685	1	Simoa
Wolf et al. (2020) [49]	CN (4444)	42	71.9 ± 7.5	2.6 ± 2.3	1	Simoa
Verberk et al. (2020) [50]	CN (241)	60	61.9 ± 10	3.1 ± 1	1	Simoa
Pattinson et al. (2020) [51]	CN (18)	85.3	35.56 ± 12.39	2.57 ± 1.01	1	Simoa
Romero et al. (2020) [52]	CN (3472)	46	54.9 ± 13.2	3.9 ± 1.11	1	Simoa
Petersen et al. (2020) [53]	CN (225)	51.5	45.7 ± 7.1	2.4 ± 1.8	1	Simoa
Cantero et al. (2020) [54]	CN (57)	47.4	67.7 ± 3.4	3.1 ± 1.5	1	Simoa
Zetterberg et al. (2013) [55]	CN (25)	24	74 ± 6.7	4.43 ± 2.83	1 and 2	Simoa
	AD (54)	31.5	75 ± 6.2	8.80 ± 10.1		
Mattsson et al. (2016) ADNI [56]	CN (189)	55	75.9 ± 4.9	2.58 ± 1.19	1 and 2	Simoa
	AD (179)	52	75.2 ± 7.4	3.12 ± 1.50		
Mattsson et al. (2016) BioFINDER [56]	CN (274)	39	72.9 ± 4.9	5.58 ± 2.51	1 and 2	Simoa
	AD (61)	42	76.4 ± 4.7	5.37 ± 2.56		
Shi et al. (2016) [44]	CN (106)	54.7	67.1 ± 7.4	2.405 ± 2.76	1, 2 and 3	Simoa
	AD (106)	53.8	69.5 ± 8.1	3.870 ± 2.22		
Kovacs et al. (2017) [57]	CN (18)	50	73.7	1.68 ± 0.17	1 and 2	Simoa
	AD (21)	23.8	77	7.5 ± 3.2		
Deters et al. (2017) [58]	CN (166)	57.2	75.2 ± 5.1	2.71 ± 1	1 and 2	Simoa
	AD (168)	51.8	75.3 ± 7.3	3.13 ± 1.3		
Chen et al. (2017) [59]	CN (151)	58.9	75.7 ± 4.9	2.7 ± 1.1	1 and 2	Simoa
	AD (149)	55.7	76.1 ± 7.3	3.2 ± 1.3		
Mielke et al. (2018) [60]	CN (172)	69.2	71.9 ± 9.5	5.9 ± 1.90	1 and 2	Simoa
	AD (40)	23	67.7 ± 9.2	7.2 ± 2.80		
Park et al. (2019) [61]	CN (172)	40.4	71.08 ± 1.0	2.37 ± 0.1	1, 2 and 3	Simoa
	AD (40)	13.3	75.87 ± 2.1	3.36 ± 0.3		
Li et al. (2019) [62]	CN (9)	44.4	61.78 ± 10.52	4.62 ± 0.50	1 and 2	Simoa
	AD (53)	42.3	68.39 ± 9.65	5.47 ± 2.69		
Startin et al. (2019) [63]	CN (27)	59.3	49.26 ± 10.40	1.49 ± 1.26	1 and 2	Simoa
	AD (27)	66.7	59.33 ± 4.04	1.45 ± 1.02		
Sugarman et al. (2020) [64]	CN (238)	37.4	72.38 ± 7.69	3.22 ± 2.73	1, 2 and 3	Simoa
	AD (156)	55.8	76.74 ± 8.12	3.73 ± 3.01		
Fossati et al. (2020) [65]	CN (68)	35.3	67.71 ± 8.54	2.74 ± 0.76	1, 2 and 3	Simoa
	AD (29)	34.5	72.81 ± 9.69	3.67 ± 1.06		
Deniz et al. (2020) [66]	CN (162)	23.5	82.7 ± 8.15	3.84 ± 2.27	1 and 2	Simoa
	AD (159)	31.2	78.2 ± 8.97	3.75 ± 2.36		
Lin et al. (2018) [67]	CN (35)	40.0	62.6 ± 9.7	12.12 ± 0.96	1	IMR
Chi et al. (2019) [68]	CN (42)	79	59 ± 11.1	18.2 ± 13.78	1	IMR
Chen et al. (2019) [69]	CN (13)	69.2	73 ± 13.0	18.7 ± 5.63	1	IMR
Chen et al. (2020) [70]	CN (28)	28.6	61.1 ± 4.9	14.67 ± 8.49	1	IMR
Fang et al. (2020) [71]	CN -young (43)	67	38.7 ± 13.5	14.9 ± 5.5	1	IMR
	CN -old (34)	41	70.3 ± 5.8	15.0 ± 7.3	1	
Chiu et al. (2017) [72]	CN- middle (56)	29.1	58.1 ± 4.9	14.35 ± 6.49	1	IMR
	CN- old (70)	45.7	73.6 ± 6.3	18.14 ± 7.33	1	
Chiu et al. (2013) [73]	CN (30)	43.3	64.4 ± 9.5	15.6 ± 6.9	1 and 2	IMR
	AD (10)	40	69.3 ± 9.4	53.9 ± 11.7		
Tzen et al. (2014) [74]	CN (20)	50	63.7 ± 7.9	13.5 ± 5.5	1 and 2	IMR
	AD (14)	28.6	64.9 ± 11.5	46.7 ± 2.0		
Yang et al. (2017) [75]	CN (66)	NA	64.6 ± 8.6	13.37 ± 7.77	1 and 2	IMR
	AD (29)		72.2 ± 9.9	55.44 ± 22.45		
Lee et al. (2017) [76]	CN-young (44)	68.1	38.4 ± 13.5	14.9 ± 5.5	1 and 2	IMR
	CN-old (34)	41.2	70.3 ± 5.8	15.0 ± 7.3		
	AD (62)	46.8	72.1 ± 11.1	47.5 ± 18.9		
Lue et al. (2017) BSHRI [77]	CN (16)	25	81.9 ± 1.5	20.48 ± 1.24	1 and 2	IMR
	AD (16)	43.8	82.5 ± 1.4	34.52 ± 3.75		
Lue et al. (2017) NTUH [77]	CN (61)	39.3	64.2 ± 1.1	13.98 ± 1.89	1 and 2	IMR
	AD (31)	54.8	72.5 ± 1.8	52.47 ± 2.72		
Yang et al. (2018) [78]	CN (23)	NA	67.5 ± 7.1	18.85 ± 10.16	1 and 2	IMR
	AD (21)		78.8 ± 7.9	37.54 ± 12.29		
Chiu et al. (2019) BSHRI [79]	CN (16)	NA	81.9 ± 6	20.48 ± 4.96	1 and 2	IMR
	AD (16)		82.5 ± 1.4	34.52 ± 14		
Chiu et al. (2019) NTUH [79]	CN (37)	NA	66.1 ± 8.3	16.61 ± 9.18	1 and 2	IMR
	AD (25)		78.1 ± 7.3	43.35 ± 15.14		
Jiao et al. (2020) [80]	CN (57)	45.6	67.9 ± 9.5	20.65 ± 3.52	1 and 2	IMR
	AD (40)	37.5	68.1 ± 9.0	25.91 ± 8.12		
Liu et al. (2020) [81]	CN (2)	50	37 ± 2.8	17.4 ± 1.1	1 and 2	IMR
	AD (3)	33.3	71.3 ± 4.0	28.3 ± 4.0		
Rubenstein et al. (2017) [82]	CN (20)	70	40.5 ± 14.2	0.063 ± 0.014	1	a-EIMAF
Gardner et al. (2018) [83]	CN-young (79)	73.4	25.8 ± 7.3	0.079 ± 0.0087	1	a-EIMAF
	CN-middle-aged (60)	66.7	50.0 ± 5.9	0.078 ± 0.008		
	CN-older (30)	63.3	68.0 ± 8.4	0.079 ± 0.0065

**Table 2:** Basic characteristics of included studies for plasma ptau181 analysis

Study	Subjects (***n***)	Male (%)	Age, years (mean ± SD)	P-tau-181 (pg/ml)	Research question	Method
Suárez-Calvet et al. (2020) [84]	CN (250)	38	60.6 ± 4.44	8.83 ± 3.21	1	Simoa - Karikari method
Moscoso et al. (2021) [85]	CN (374)	47.1	74.8 ± 6.6	13.3 ± 10.7	1	Simoa - Karikari method
O’Connor et al. (2020) [86]	CN (27)	41	38.1 ± 10.7	9.7 ± 9.3	1, 2 and 3	Simoa - Karikari method
	AD (19)	63	50.7 ± 10.0	23.7 ± 10.5		
Rodriguez et al. (2020) [87]	CN (28)	35.7	82.2 ± 6.5	19.3 ± 9.9	1, 2 and 3	Simoa - Karikari method
	AD (77)	46.1	81.7 ± 7.6	28.4 ± 9.6		
Karikari et al. (2020) - TRIAD cohort [88]	CN-young (27)	37	22.7 ± 1.9	7.9 ± 2.6	1, 2 and 3	Simoa - Karikari method
	CN-old (113)	36	69.2 ± 9.7	10 ± 3.3		
	AD (33)	55	64.6 ± 9.2	24.9 ± 7.8		
Karikari et al. (2020) -BioFINDER-2 cohort [88]	CN-old (337)	46	63.1 ± 5.0	9.4 ± 6.0	1, 2 and 3	Simoa - Karikari method
	AD (126)	47	74.0 ± 6.9	19.2 ± 9.4		
Karikari et al. (2020) [45]	CN (268)	51.1	73.5 ± 6.5	14.2 ± 9.0	1, 2 and 3	Simoa - Karikari method
	AD (137)	52.2	73.4 ± 8.2	25.8 ± 8.6		
Mielke et al. (2018) [60]	CN (172)	69.2	71.9 ± 9.5	6.4 ± 6.4	1, 2 and 3	MSD
	AD (40)	23	67.7 ± 9.2	11.6 ± 4.1		
Thijssen et al. (2020) [89]	CN (69)	53.6	60.8 ± 22	2.4 ± 3	1, 2 and 3	MSD
	AD (56)	41.1	65 ± 9	8.4 ± 4		
Janelidze et al. (2020) - Cohort1 [90]	CN (26)	38.5	74 ± 5.2	1.3 ± 1.1	1, 2 and 3	MSD
	AD (38)	44.7	73 ± 8.1	4.4 ± 2.3		
Janelidze et al. (2020) - Cohort2 [90]	CN (126)	61.9	71 ± 5.2	1.2 ± 0.59	1, 2 and 3	MSD
	AD (81)	43.2	73 ± 5.2	2.8 ± 2.07		
Janelidze et al. (2020) – Cohort3 [90]	CN (47)	59.6	83 ± 9.6	1.8 ± 1.04	1, 2 and 3	MSD
	AD (16)	75.0	83 ± 7.4	4 ± 2.07		

### Plasma tau and ptau181 levels in healthy cohorts

Subgroup analysis was performed according to age and sex ratio, among studies using Simoa, IMR, a-EIMAF, and MSD, respectively. Based on the data retrieved, the average age of cohort stratification was classified as young (< 40 years old), middle-aged (40–60 years old), and old (> 60 years). The sex ratio was defined as low (less than 40% male), middle (40%–60% male), and high (over 60% male).

**Fig. 2:**
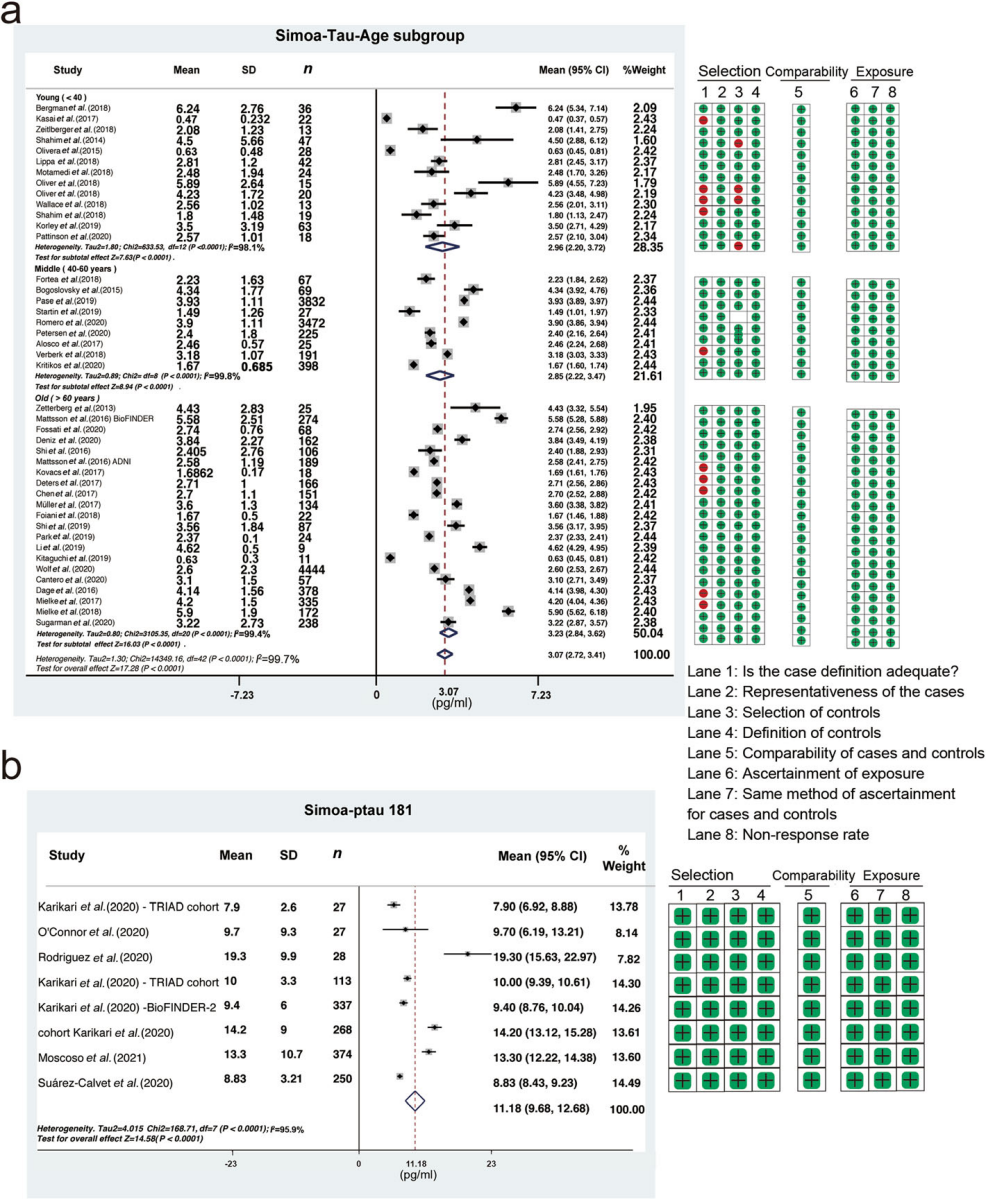
The levels of plasma tau and ptau181 in healthy people detected by the Simoa method. Meta-analysis of studies calculating plasma tau levels (**a**) and plasma ptau181 levels (**b**) of different age groups in the healthy population. In ptau181 studies, the average age in Rodriguez et al. was considerably higher; the control cohort used in Suarez-Calvet et al. comprised ‘middle-aged’ adults; the TRIAD cohort in Karikari et al. included young individuals; all of which may have contributed to the heterogeneity

Among the 66 studies, 58 studies that used Simoa, IMR, or a-EIMAF were included for the analysis of normal range of plasma tau, as the MSD studies focused on CSF tau. Forty-one publications, reporting 42 cohorts, were retrieved using the Simoa technology [8, 27–29, 31–44, 46–49, 51–66, 91–93]. Our results indicated that the average plasma tau level in healthy populations was 3.07 pg/ml (95% CI 2.72–3.41, *I*^2^ = 99.7%, *P*  < 0.0001). The plasma tau levels in the young, middle-aged, and old groups were 2.96 pg/ml (95% CI 2.20–3.72), 2.85 pg/ml (95% CI 2.22–3.47), and 3.23 pg/ml (95% CI 2.84–3.62), respectively (Fig. 2a), without significant differences (*P* = 0.099). However, significant differences were identified in the subgroup analysis by sex ratio (4.15 pg/ml *vs* 2.64 pg/ml *vs* 3.24 pg/ml; *P* < 0.0001; Fig. S2). Fifteen publications [67–81] reporting 20 cohorts were retrieved using IMR and two publications [82, 83] reporting four cohorts were retrieved using a-EIMAF. The effect sizes (ESs) for plasma tau levels were 16.30 pg/ml (95% CI 14.61–17.99, *I*^2^ = 92.7%, *P* < 0.0001, Fig. S3) and 76.14 fg/ml (95% CI 72.34–79.93, *I*^2^ = 88.8%, *P* < 0.0001, Fig. S4), respectively.

Nine studies that used Simoa or MSD methods were included for the analysis of normal range of plasma ptau181. Six publications, reporting seven cohorts, were retrieved using the Simoa technology [45, 84–88], including 1424 healthy subjects. The ES for plasma ptau181 levels in healthy populations was 11.18 pg/ml (95% CI 9.68–12.68, *I*^2^ = 95.9%, *P* < 0.0001, Fig. 2b). There were no significant differences in plasma ptau181 level in the subgroup analysis concerning age (*P* = 0.181) or the sex ratio (*P* = 0.168, Fig. S2). Three publications [60, 89, 90] reporting five cohorts using the MSD method, including 440 healthy subjects who could not be grouped by age or sex ratio, were identified. The ES for plasma ptau181 levels measured by MSD was 2.48 pg/ml (95% CI 1.57–3.37, *I*^2^ = 97.0%, *P* < 0.0001; Fig. S4).

### Plasma total tau and ptau181 in AD patients and controls

**Fig. 3:**
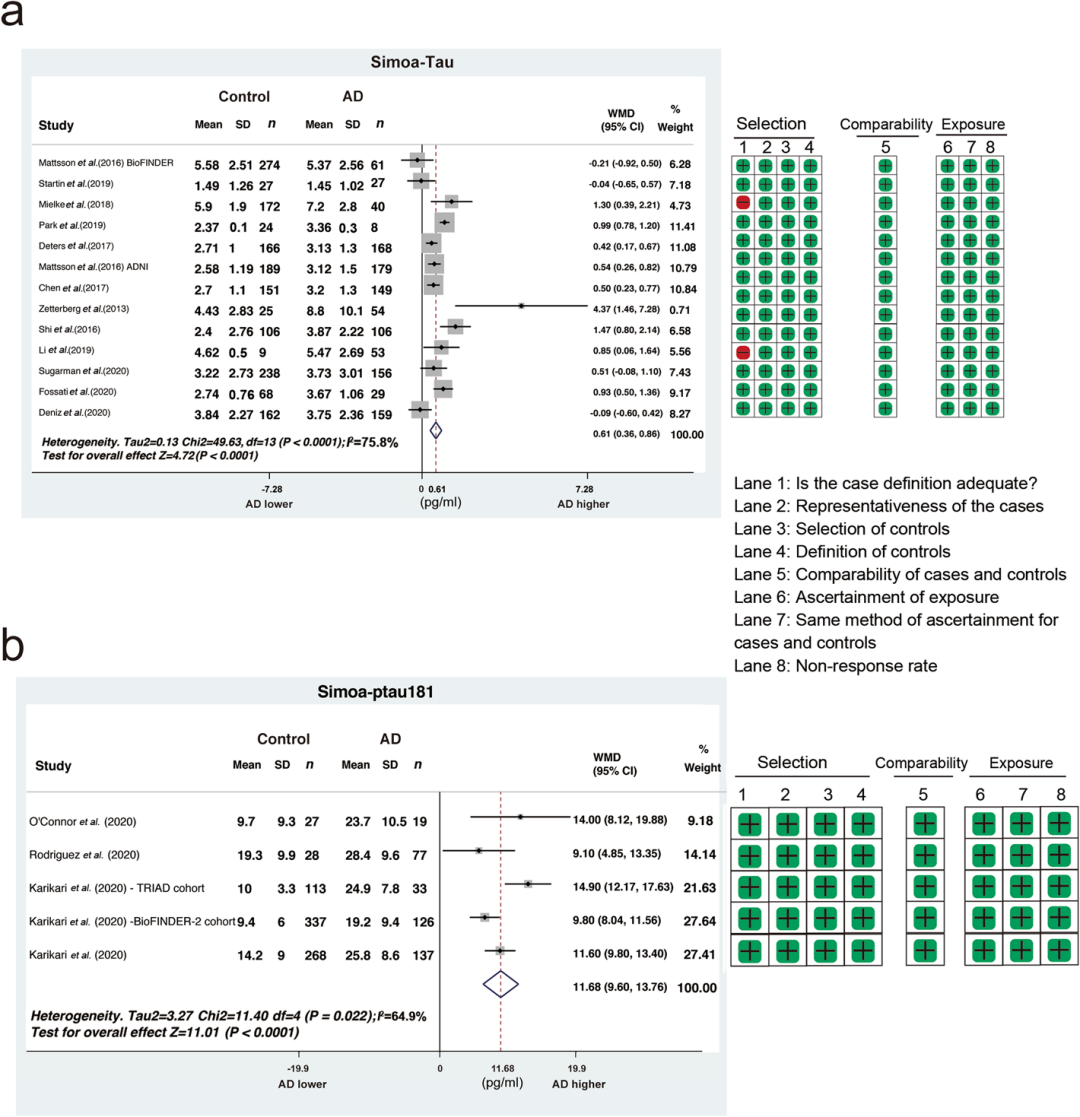
Comparison of plasma tau or ptau181 between AD and healthy controls. Meta-analysis of studies comparing plasma tau (**a**) and ptau181 (**b**) levels between AD and healthy controls, detected by the Simoa method. In ptau181 studies, the study of O’connor et al. was a familial AD study; the average age in the study of Rodriguez et al. was considerably higher; the control cohort used in the study of Suarez-Calvet et al. comprised ‘middle-aged’ adults; the TRIAD cohort in Karikari et al. included young individuals, all of which may have contributed to the heterogeneity. AD, Alzheimer’s disease; SD, standard deviation; CI, confidence interval; WMD, weighted mean difference

We then compared plasma tau and ptau181 between AD and controls. The random-effects model of meta-analysis with subgroup analysis was performed according to age and sex ratio. Among the 66 studies, 22 studies that used Simoa and IMR were included for the analysis of difference of plasma tau between AD and controls, and the number of studies that used other technologies was insufficient for analysis. For the Simoa method, 13 papers, reporting 14 cohorts [44, 55–66], were retrieved with a total of 1189 AD patients with 1611 controls. Our analysis revealed a significantly higher plasma tau level in patients with AD, with an average WMD value of 0.61 (95% CI 0.36–0.86, *I*^2^ = 75.8%, *P* < 0.0001; Fig. 3a). The WMDs for subgroups divided by different male compositions were 0.48 (< 40% male, 95% CI − 0.18–1.14), 0.99 (40%–60% male, 95% CI 0.54–1.44) and 1.30 (> 60% male, 95% CI 0.39–2.21), respectively, indicating a strong effect of sex on plasma tau levels in AD patients (Fig. S5). Meta-regression analysis identified that only the sample size significantly contributed to the high heterogeneity (*P* = 0.027, Table S2). The funnel plots and the Egger’s test suggested no publication bias (*P* = 0.133) (Fig. S6). Similarly, 9 papers [72–74, 76–81] reporting 11 cohorts using the IMR method (362 controls and 267 patients) were identified (Fig. S5), showing an average WMD value of 24.83 (95% CI 15.70–33.96, *I*^2^ = 98.9%, *P* < 0.0001). There was no publication bias (*P* = 0.175, Fig. S6), but the year of publication (*P* = 0.013) may influence the heterogeneity (Table S2).

Seven studies that used Simoa and MSD were included for the analysis of the plasma ptau181 difference between AD and controls. Four publications reporting five cohorts [45, 86–88] using the Simoa technology were retrieved, resulting in a total of 773 controls and 392 patients. The plasma ptau181 levels were significantly elevated in AD patients, with an average WMD value of 11.68 (95% CI 9.60–13.76, *I*^2^ = 64.9%, *P* < 0.0001, Fig. 3b). The funnel plots suggested no publication bias for plasma ptau181 (*P* = 0.635, Fig. S6). Similar results were found based on three papers reporting five cohorts [60, 89, 90] using the MSD method (440 controls and 231 patients), with a WMD of 3.53 (95% CI 1.97–5.09, *I*^2^ = 93.0%, *P* < 0.0001, Fig. S5). There was also no publication bias (*P* = 0.055, Fig. S6).

### The diagnostic accuracy of plasma tau/ptau181 for AD

**Fig. 4:**
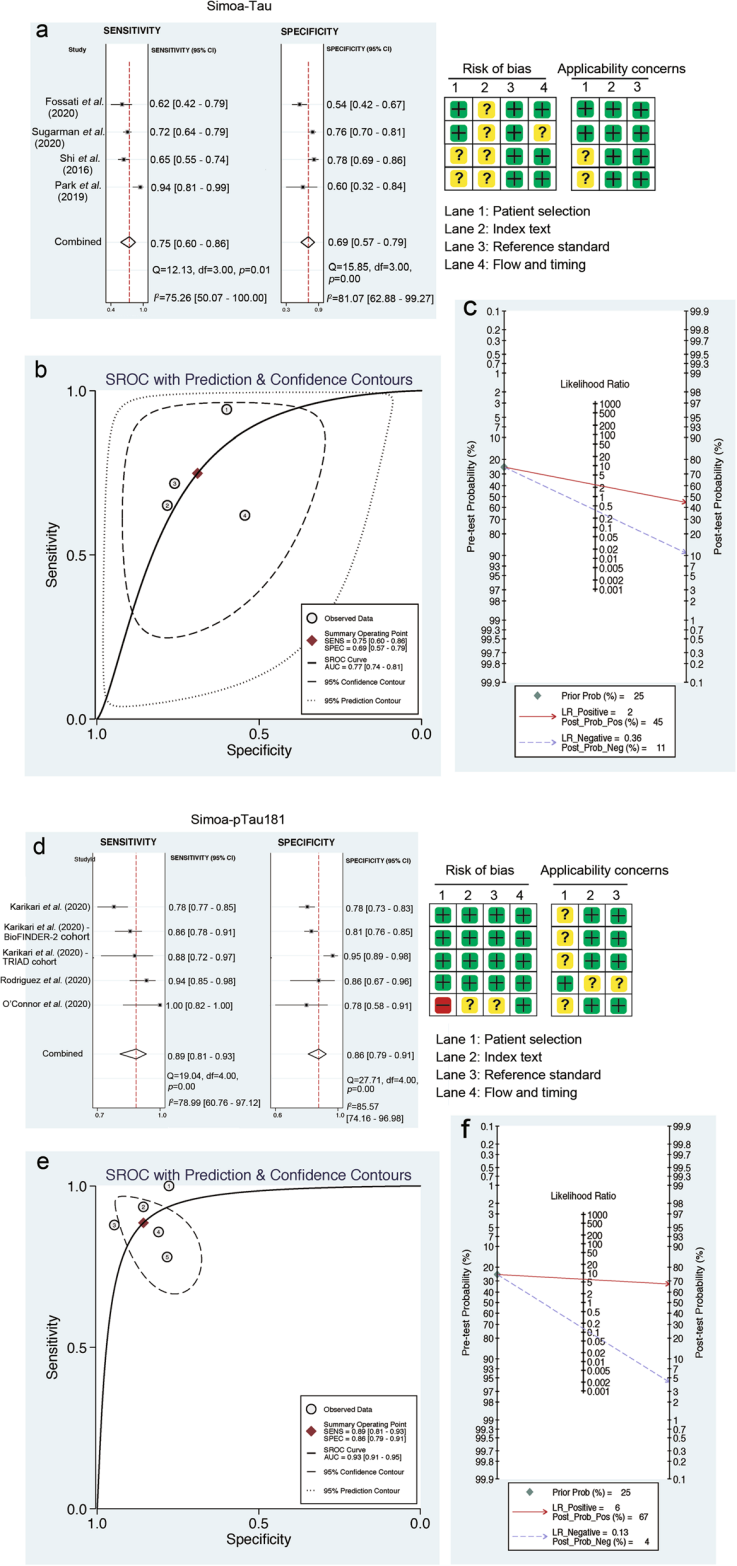
Diagnostic accuracy of plasma tau and ptau181. **a–c** Forest plots of pooled sensitivity and specificity (**a**), HSROC curve (**b**), and Fagan’s nomogram (**c**) to estimate the clinical utility of plasma tau detected by the Simoa method. **d–f** The forest plots of pooled sensitivity and specificity (**d**), HSROC curve (**e**) and Fagan’s nomogram (**f**) to estimate the clinical utility of plasma ptau181 detected by the Simoa method

Among the 66 studies, four studies reporting five cohorts [44, 61, 64, 65] were pooled for meta-analysis of diagnostic accuracy of plasma tau using the Simoa method. The estimate values of diagnostic accuracy are summarized in Table S3. The pooled sensitivity and specificity of plasma tau to predict AD were 0.75 (95% CI 0.60–0.86) and 0.69 (95% CI 0.57–0.79), respectively (Fig. 4a). The DOR was 6.16 (95% CI 3.02–12.53), and the AUC of the HSROC curve was 0.77 (95% CI 0.74–0.81) (Fig. 4b). Fagan’s nomogram showed that the probability of AD increased from 25% (pre-test probability) to 45% (post-test probability) when plasma tau level was above the threshold, while the probability of AD decreased to as low as 11% when plasma tau level was below the threshold (Fig. 4c).

Four studies using the Simoa method reporting five cohorts [45, 86–88] were pooled for meta-analysis to test the diagnostic accuracy of plasma ptau181. The estimate values of diagnostic accuracy are summarized in Table S3. The pooled sensitivity and specificity of ptau181 were 0.89 (95% CI 0.81–0.93) and 0.86 (95% CI 0.79–0.91), respectively (Fig. 4d). The DOR was 46 (95% CI 18–123), and the AUC of the HSROC curve was 0.93 (95% CI 0.91–0.95) (Fig. 4e). The probability of AD increased from 25% (pre-test probability) to 67% (post-test probability) when plasma ptau181 level was above the threshold and decreased to as low as 4% when plasma ptau181 level below the threshold (Fig. 4f). Consistently, two studies using the MSD method reporting four cohorts [89, 90] were pooled for meta-analysis. The estimate values of diagnostic accuracy are summarized in Table S3. The pooled sensitivity of ptau181 was 0.87 (95% CI 0.78–0.92), and the pooled specificity was 0.79 (95% CI 0.73–0.83, Fig. S7). The DOR was 23.98 (95% CI 10.14–56.69), and the AUC of the HSROC curve was 0.86 (95% CI 0.83–0.89, Fig. S7). The probability of AD increased from 25% (pre-test probability) to 58% (post-test probability) when plasma ptau181 level was above the threshold, and decreased to 5% when plasma ptau181 level was below the threshold (Fig. S7).

## Discussion

With a growing interest in plasma tau detection during the last 10 years, heterogeneity between studies has been consistently presented. Besides, given the inconsistency between publications regarding the plasma tau levels in AD (compared to control), it is difficult to determine the suitability of plasma tau/ptau181 to predict AD. In this meta-analysis, we found that both plasma tau and ptau181 have diagnostic values, and both of them are significantly higher in AD patients than in controls. We also established the average plasma tau and ptau181 levels based on the current literature, which may be used as a reference point in future research.

Currently, there are four ultrasensitive assays that can be used for plasma tau and ptau detection. In this meta-analysis, we examined them independently. Despite the differences in absolute values of plasma tau and ptau, results from all assays support the notion that both plasma tau and ptau181 are elevated in AD compared with healthy controls. Based on the available publications of each method, we have been able to calculate the sensitivity and diagnostic accuracy for AD of studies using Simoa (tau AUC: 0.77; ptau181 AUC: 0.93), and MSD methods (ptau181 AUC: 0.86), while the other two methods had limited applications in AD research.

Both Aβ and tau can now be visualized in the brain using PET and be measured in the CSF. Although medical history and cognitive and neurologic examinations remain the most important diagnostic tool in the clinic [94], these new techniques can assist the diagnosis of AD [1]. However, the associated cost and infrastructure requirements have limited their use, especially in developing countries. It is critical to accurately measure AD-associated proteins in plasma and determine their relationships with brain and CSF contents. Proteins in plasma may reflect protein levels in the brain and CSF, especially in the state of illness. For example, the correlation between CSF and plasma ptau181 is significant in PET Aβ-positive cases, even without cognitive impairment [90]. Higher ptau181 is associated with increased standardized uptake value ratio of tau PET in Braak I–IV regions of interest [89]. Furthermore, our meta-analysis results reflected the high diagnostic accuracy of plasma tau (AUC 0.77, 95% CI 0.74–0.81) and ptau181 (AUC 0.93, 95% CI 0.91–0.95), similar to the diagnostic accuracy of tau PET (AUC 0.98, 95% CI 0.94–1.00) and CSF ptau181 (AUC 0.97, 95% CI 0.92–1.00) [90]. In contrast, there is only a weak association between CSF and plasma tau, as confirmed by two independent studies [46, 65], suggesting that they may go through different metabolism. There are no significant differences in plasma or serum Aβ between AD and controls, and plasma Aβ may reflect peripheral Aβ generation instead of brain pathology [95].

In addition, the current meta-analysis supports plasma ptau181 as a better predictive biomarker than plasma tau for AD. The difference in plasma ptau181 between AD and controls was greater than plasma tau in our analysis (WMD: 11.68 pg/ml *vs* 0.83 pg/ml), and the pooled diagnostic accuracy of ptau181 was also higher (AUC: 0.93 *vs* 0.77). These are consistent with a previous report that the plasma ptau181 is more strongly associated with both Aβ and tau PET than plasma tau [60], and can differentiate AD from non-AD pathologies with high accuracy (AUC 97.4, 95% CI 94.1%–100%) eight years before death [87], collectively highlighting the potential of ptau181 as a biomarker for AD pathology.

We also found a higher level of plasma ptau181 compared to total tau in the meta-analysis. Phosphorylation is a post-translational modification of the protein, and theoretically, ptau181 should be a portion of total tau in any given tissue [96]. However, according to our analysis of publications based on the Simoa method, the mean level of ptau181 was higher than total tau (11.18 pg/ml *vs* 3.07 pg/ml). There could be differences in calibration standard, and at this stage, it is not feasible to compare the calculated values of tau and ptau181.

### Limitations

There were some limitations in this systematic review and meta-analysis. The ultrasensitive measurement of plasma tau has yet to be extensively tested, leading to high heterogeneity in the results. Meta-regression analyses suggested that the heterogeneity was partly explained by the sample size of studies included. In addition, most of the studies included in the current analysis used clinical diagnostic guidelines rather than gold-standard autopsy-confirmed AD, which can induce heterogeneity. One study [57] on neuropathologically confirmed autopsy cases has revealed a greater change in plasma tau (WMD 5.81), compared to the results from the clinically diagnosed cases (WMD 0.83), highlighting the potential error induced by diagnosis. Even the diagnosis is correct, AD itself can be heterogenetic. For example, familial cohorts [86] may lead to different results from sporadic cohorts.

On the other hand, due to the lack of information on ApoE4 status, we could not measure the impact of ApoE on plasma tau and ptau, leading to potential heterogeneity. We have considered age as a factor for heterogeneity, but due to the limited data, we can only analyze the effect of age in healthy populations, where we found no effect of age on plasma tau. Future analysis should be performed when there are more studies on early-onset AD.

We excluded a few publications for analysis. Two using the Simoa platform were excluded as the methods used in those studies had not been validated in multiple cohorts or studies [97], or were specifically validated for CSF (not plasma) [61]. Although results of the two studies are consistent with our findings, it is challenging to combine them with those using the commercialized plasma tau detection kit developed by Quanterix. A few other studies were focused on the serum tau or ptau [98, 99], and were also excluded from our analysis.

We have to mention that some studies on ptau181 were recently published during the final revision of the manuscript, and were not included. These latest studies have investigated the dynamic changes of plasma ptau181 across the AD spectrum [100] and the relationship between polygenic risk scores for AD and plasma ptau181 [101]. They have also compared performance of ptau181 with other biomarkers in AD and MCI prediction [102] or amyloid PET status prediction [103]. In addition, ptau217 [104] and ptau231 (Ashton et al. in press) have been reported recently to differentiate AD from other neurodegenerative disorders and be associated with tau pathology in the brain. The plasma ptau217 may out-perform ptau181 with a higher AUC and stronger correlations with the tau PET [9, 104]. However, here we did not include plasma ptau217 and ptau231 studies in our meta-analysis, due to the limited number of publications.

## Conclusion

In summary, the results presented provide preliminary evidence of plasma tau and its phosphorylated form ptau181 as potential biomarkers for neurological diseases, especially for AD diagnosis, which may facilitate drug discovery of these diseases by selecting correct patients for clinical trials.

## Additional Files

**Additional file 1 Table S1.** The Newcastle-Ottawa Scale (NOS) for assessing the quality of studies in meta-analyses. **Table S2.** Meta-regression results. **Table S3.** Summary of estimate values of diagnostic accuracy.

**Additional file 2 Fig. S1.** Quality assessment results of included articles. **Fig. S2.** The levels of plasma tau and ptau181 in healthy people using Simoa in different subgroups. **Fig. S3.** The level of plasma tau in healthy people using IMR in different subgroups. **Fig. S4.** The levels of plasma tau and ptau181 in healthy people using EIMAF/a-EIMAF and MSD. **Fig. S5.** Comparison of plasma tau and ptau181 between AD and healthy controls using Simoa, IMR, and MSD. **Fig. S6.** Funnel plot of the random-effect analysis. **Fig. S7.** The diagnostic accuracy of plasma ptau181 using MSD.

## Data Availability

The datasets supporting the conclusions of this article are included within the article and its additional files.

## Abbreviations

AD: Alzheimer’s disease
ptau181: Phosphorylated tau 181
AUC: Area under curve
ROC: Receiver operating characteristic
NIA-AA: National Institute on Aging—Alzheimer’s Association
Aß: Amyloid-beta
PET: Positron emission tomography
NFT: Neurofibrillary tangle
CSF: Cerebrospinal fluid
Simoa: Single-molecule Array
IMR: ImmunoMagnetic Reduction
a-EIMAF: multi-arrayed fiber optics conjugated with rolling circle amplification
MSD: Meso Scale Discovery
NOS: Newcastle-Ottawa Scale
QUADAS-2: Quality Assessment of Diagnostic Accuracy Studies-2 tool
WMD: Weighted mean difference
DOR: Diagnostic odds ratio
HSROC: Hierarchical summary receiver operating characteristic
