# Supplementary material for: Ultrasensitive assays for detection of plasma tau and phosphorylated tau 181 in Alzheimer’s disease: a systematic review and meta-analysis
Source: Transl Neurodegener. 2021 Mar 12;10:10. doi: 10.1186/s40035-021-00234-5 (PMC7953695; doi:10.1186/s40035-021-00234-5)
Supplement: Supplementary file 1 — Additional file 1 Table S1. The Newcastle-Ottawa Scale (NOS) for assessing the quality of studies in meta-analyses. Table S2. Meta-regression results. Table S3. Summary of estimate values of diagnostic accuracy. [file 40035_2021_234_MOESM1_ESM.docx]

## Table S1. The Newcastle-Ottawa Scale (NOS) for assessing the quality of studies in meta-analyses

| Studies |  | Selection | | | |  | Comparability |  | Exposure | | |  | Total Quality score |
| --- | --- | --- | --- | --- | --- | --- | --- | --- | --- | --- | --- | --- | --- |
| Author, year |  | Is the Case Definition Adequate? | Representativeness of the Cases | Selection of Controls | Definition of Controls |  | Comparability of cases and controls |  | Ascertainment of exposure | Same method of ascertainment for cases and controls | Non-Response rate |  |  |
| Bogoslovsky et al.(2015) |  | 1 | 1 | 1 | 1 |  | 1 |  | 1 | 1 | 1 |  | 8 |
| Mielke et al.(2018) |  | 1 | 1 | 1 | 1 |  | 1 |  | 1 | 1 | 1 |  | 8 |
| Park et al.(2019) |  | 1 | 1 | 1 | 1 |  | 1 |  | 1 | 1 | 1 |  | 8 |
| Pase et al.(2019) |  | 1 | 1 | 1 | 1 |  | 1 |  | 1 | 1 | 1 |  | 8 |
| Kovacs et al.(2017) |  | 0 | 1 | 1 | 1 |  | 1 |  | 1 | 1 | 1 |  | 7 |
| Deters et al.(2017) |  | 1 | 1 | 1 | 1 |  | 1 |  | 1 | 1 | 1 |  | 8 |
| Alosco et al.(2017) |  | 1 | 1 | 1 | 1 |  | 1 |  | 1 | 1 | 1 |  | 8 |
| Dage et al.(2016) |  | 1 | 1 | 1 | 1 |  | 1 |  | 1 | 1 | 1 |  | 8 |
| Mielke et al.(2017) |  | 1 | 1 | 1 | 1 |  | 1 |  | 1 | 1 | 1 |  | 8 |
| Müller et al.(2017) |  | 1 | 1 | 1 | 1 |  | 1 |  | 1 | 1 | 1 |  | 8 |
| Kasai et al.(2017) |  | 1 | 1 | 1 | 1 |  | 1 |  | 1 | 1 | 1 |  | 8 |
| Mattsson et al.(2016) |  | 1 | 1 | 1 | 1 |  | 1 |  | 1 | 1 | 1 |  | 8 |
| Foiani et al.(2018) |  | 1 | 1 | 1 | 1 |  | 1 |  | 1 | 1 | 1 |  | 8 |
| Chen et al.(2017) |  | 1 | 1 | 1 | 1 |  | 1 |  | 1 | 1 | 1 |  | 8 |
| Bergman et al.(2018) |  | 1 | 1 | 1 | 1 |  | 1 |  | 1 | 1 | 1 |  | 8 |
| Petersen et al.(2020) |  | 1 | 1 | 1 | 1 |  | 1 |  | 1 | 1 | 1 |  | 8 |
| Cantero et al.(2020) |  | 1 | 1 | 1 | 1 |  | 1 |  | 1 | 1 | 1 |  | 8 |
| Deniz et al.(2020) |  | 1 | 1 | 1 | 1 |  | 1 |  | 1 | 1 | 1 |  | 8 |
| Lippa et al.(2018) |  | 1 | 1 | 1 | 1 |  | 1 |  | 1 | 1 | 1 |  | 8 |
| Shi et al.(2016) |  | 1 | 1 | 1 | 1 |  | 1 |  | 1 | 1 | 1 |  | 8 |
| Wallace et al.(2018) |  | 0 | 1 | 1 | 1 |  | 1 |  | 1 | 1 | 1 |  | 7 |
| Korley et al.(2019) |  | 1 | 1 | 0 | 1 |  | 1 |  | 1 | 1 | 1 |  | 7 |
| Zetterberg et al.(2013) |  | 1 | 1 | 1 | 1 |  | 1 |  | 1 | 1 | 1 |  | 8 |
| Shahim et al.(2014) |  | 1 | 1 | 1 | 1 |  | 1 |  | 1 | 1 | 1 |  | 8 |
| Verberk et al.(2018) |  | 1 | 1 | 1 | 1 |  | 1 |  | 1 | 1 | 1 |  | 8 |
| Shahim et al.(2018) |  | 0 | 1 | 1 | 1 |  | 1 |  | 1 | 1 | 1 |  | 7 |
| Olivera et al.(2015) |  | 1 | 1 | 1 | 1 |  | 1 |  | 1 | 1 | 1 |  | 8 |
| Shi et al.(2019) |  | 1 | 1 | 1 | 1 |  | 1 |  | 1 | 1 | 1 |  | 8 |
| Kitaguchi et al.(2019) |  | 0 | 1 | 1 | 1 |  | 1 |  | 1 | 1 | 1 |  | 7 |
| Li et al.(2019) |  | 0 | 1 | 1 | 1 |  | 1 |  | 1 | 1 | 1 |  | 7 |
| Startin et al.(2019) |  | 1 | 1 | 1 | 1 |  | 1 |  | 1 | 1 | 1 |  | 8 |
| Motamedi et al.(2018) |  | 1 | 1 | 1 | 1 |  | 1 |  | 1 | 1 | 1 |  | 8 |
| Fortea et al.(2018) |  | 1 | 1 | 1 | 1 |  | 1 |  | 1 | 1 | 1 |  | 8 |
| Zeitlberger et al.(2018) |  | 0 | 1 | 1 | 1 |  | 1 |  | 1 | 1 | 1 |  | 7 |
| Sugarman et al.(2020) |  | 1 | 1 | 1 | 1 |  | 1 |  | 1 | 1 | 1 |  | 8 |
| Kritikos et al.(2020) |  | 1 | 1 | 1 | 1 |  | 1 |  | 1 | 1 | 1 |  | 8 |
| Wolf et al.(2020) |  | 1 | 1 | 1 | 1 |  | 1 |  | 1 | 1 | 1 |  | 8 |
| Verberk et al.(2020) |  | 1 | 1 | 1 | 1 |  | 1 |  | 1 | 1 | 1 |  | 8 |
| Fossati et al.(2020) |  | 1 | 1 | 1 | 1 |  | 1 |  | 1 | 1 | 1 |  | 8 |
| Pattinson et al.(2020) |  | 0 | 1 | 1 | 1 |  | 1 |  | 1 | 1 | 1 |  | 7 |
| Romero et al.(2020) |  | 1 | 1 | 0 | 1 |  | 1 |  | 1 | 1 | 1 |  | 7 |
| Jiao et al.(2020) |  | 1 | 1 | 1 | 1 |  | 1 |  | 1 | 1 | 1 |  | 8 |
| Liu et al.(2020) |  | 0 | 1 | 1 | 1 |  | 1 |  | 1 | 1 | 1 |  | 7 |
| Fang et al.(2020) |  | 1 | 1 | 1 | 1 |  | 1 |  | 1 | 1 | 1 |  | 8 |
| Chen et al.(2020) |  | 1 | 1 | 1 | 1 |  | 1 |  | 1 | 1 | 1 |  | 8 |
| Chen et al.(2019) |  | 0 | 1 | 1 | 1 |  | 1 |  | 1 | 1 | 1 |  | 7 |
| Chi et al.(2019) |  | 1 | 1 | 1 | 1 |  | 1 |  | 1 | 1 | 1 |  | 8 |
| Lin et al.(2018) |  | 1 | 1 | 1 | 1 |  | 1 |  | 1 | 1 | 1 |  | 8 |
| Yang et al.(2018) |  | 1 | 1 | 1 | 1 |  | 1 |  | 1 | 1 | 1 |  | 8 |
| Chiu et al.(2017) |  | 1 | 1 | 1 | 1 |  | 1 |  | 1 | 1 | 1 |  | 8 |
| Lue et al.(2017) |  | 0 | 1 | 1 | 1 |  | 1 |  | 1 | 1 | 1 |  | 7 |
| Tzen et al.(2014) |  | 1 | 1 | 1 | 1 |  | 1 |  | 1 | 1 | 1 |  | 8 |
| Chiu et al.(2013) |  | 1 | 1 | 1 | 1 |  | 1 |  | 1 | 1 | 1 |  | 8 |
| Yang et al.(2017) |  | 1 | 1 | 1 | 1 |  | 1 |  | 1 | 1 | 1 |  | 8 |
| Chiu et al.(2019) |  | 0 | 1 | 1 | 1 |  | 1 |  | 1 | 1 | 1 |  | 7 |
| Lee et al.(2017) |  | 1 | 1 | 1 | 1 |  | 1 |  | 1 | 1 | 1 |  | 8 |
| Rubenstein et al.(2017) |  | 1 | 1 | 1 | 1 |  | 1 |  | 1 | 1 | 1 |  | 8 |
| Gardner et al.(2018) |  | 1 | 1 | 1 | 1 |  | 1 |  | 1 | 1 | 1 |  | 8 |
| O’Connor et al.(2020) |  | 1 | 1 | 1 | 1 |  | 1 |  | 1 | 1 | 1 |  | 8 |
| Rodriguez et al.(2020) |  | 1 | 1 | 1 | 1 |  | 1 |  | 1 | 1 | 1 |  | 8 |
| Suárez-Calvet et al.(2020) |  | 1 | 1 | 1 | 1 |  | 1 |  | 1 | 1 | 1 |  | 8 |
| Moscoso et al.(2021) |  | 1 | 1 | 1 | 1 |  | 1 |  | 1 | 1 | 1 |  | 8 |
| Karikari et al.(2020) |  | 1 | 1 | 1 | 1 |  | 1 |  | 1 | 1 | 1 |  | 8 |
| Karikari et al.(2020) |  | 1 | 1 | 1 | 1 |  | 1 |  | 1 | 1 | 1 |  | 8 |
| Thijssen et al.(2020) |  | 1 | 1 | 1 | 1 |  | 1 |  | 1 | 1 | 1 |  | 8 |
| Janelidze et al.(2020) |  | 1 | 1 | 1 | 1 |  | 1 |  | 1 | 1 | 1 |  | 8 |

## Table S2. Meta regression results

|  |  | Publication year | Sample size | Region | Male percentage | Age |
| --- | --- | --- | --- | --- | --- | --- |
| Simoa-tau (*n*=13) | Coef | -0.36 | -0.0098 | -0.70 | -0.023 | 1.91 |
|  | Std | 0.21 | 0.0036 | 0.63 | 0.032 | 1.43 |
|  | 95% CI | -0.95-0.13 | -0.018- -0.0014 | -2.15-0.76 | -0.097-0.050 | -1.41-5.23 |
|  | *P* | 0.13 | 0.027 | 0.30 | 0.486 | 0.22 |
| IMR-tau (*n*=10) | Coef | -4.08 | 0.13 | 6.28 |  | 10.25 |
|  | Std | 1.22 | 0.089 | 7.27 |  | 9.65 |
|  | 95% CI | -6.97- -1.19 | -0.080-0.34 | -10.90-23.47 |  | -12.56-33.06 |
|  | *P* | 0.013 | 0.187 | 0.416 |  | 0.323 |
| MSD-Ptau181 (*n*=3) | Coef |  | -0.008 | -3.71 |  |  |
|  | Std |  | 0.0031 | 0.56 |  |  |
|  | 95% CI |  | -0.021 - 0.0052 | -6.13 - -1.28 |  |  |
|  | *P* |  | 0.121 | 0.022 |  |  |

## Table S3. Summary of estimate values of diagnostic accuracy.

|  |  | Sensitivity | Specificity | DOR | AUC | Bias |
| --- | --- | --- | --- | --- | --- | --- |
| Simoa-tau (*n*=5) |  | 0.73 | 0.71 | 6.16 | 0.78 |  |
|  | 95% CI | 0.61-0.82 | 0.61-0.79 | 3.02-12.53 | 0.74-0.81 |  |
|  | *P* | 0.01 | < 0.001 | < 0.001 | 0.094 | 0.88 |
|  | *I*^2^ | 68.20% | 76.10% | 70.4% | 40% |  |
| Simoa-Ptau181 (*n*=5) |  | 0.89 | 0.86 | 46 | 0.93 |  |
|  | 95% CI | 0.81-0.93 | 0.79-0.91 | 18-123 | 0.91-0.95 |  |
|  | *P* | 0.1 | 0.61-0.79 | < 0.001 | 0.015 | 0.21 |
|  | *I*^2^ | 78.99% | 85.57% | 76.1% | 71% |  |
| MSD-Ptau181 (*n*=3) |  | 0.87 | 0.79 | 23.98 | 0.86 |  |
|  | 95% CI | 0.78-0.92 | 0.73-0.83 | 10.14-56.69 | 0.83-0.89 |  |
|  | *P* | 0.07 | 0.12 | < 0.001 | 0.409 | 0.71 |
|  | *I*^2^ | 57.60% | 47.90% | 58.40% | 0 |  |
| *Abbreviations: AUC, area under the curve; CI, confidence interval; DOR, diagnostic odds ratio;* | | | | |  |  |
