# Supplementary material for: Ultrasensitive assays for detection of plasma tau and phosphorylated tau 181 in Alzheimer’s disease: a systematic review and meta-analysis
Source: Transl Neurodegener. 2021 Mar 12;10:10. doi: 10.1186/s40035-021-00234-5 (PMC7953695; doi:10.1186/s40035-021-00234-5)
Supplement: Supplementary file 2 — Additional file 2 Fig. S1. Quality assessment results of included articles. Fig. S2. The levels of plasma tau and ptau181 in healthy people using Simoa in different subgroups. Fig. S3. The level of plasma tau in healthy people using IMR in different subgroups. Fig. S4. The levels of plasma tau and ptau181 in healthy people using EIMAF/a-EIMAF and MSD. Fig. S5. Comparison of plasma tau and ptau181 between AD and healthy controls using Simoa, IMR, and MSD. Fig. S6. Funnel plot of the random-effect analysis. Fig. S7. The diagnostic accuracy of plasma ptau181 using MSD. [file 40035_2021_234_MOESM2_ESM.docx]

**Figure S1**. Quality assessment results of included articles. The results of each included studies (a) and all the included studies (b) by QUADAS-2. QUADAS-2, quality assessment of diagnostic accuracy studies 2.


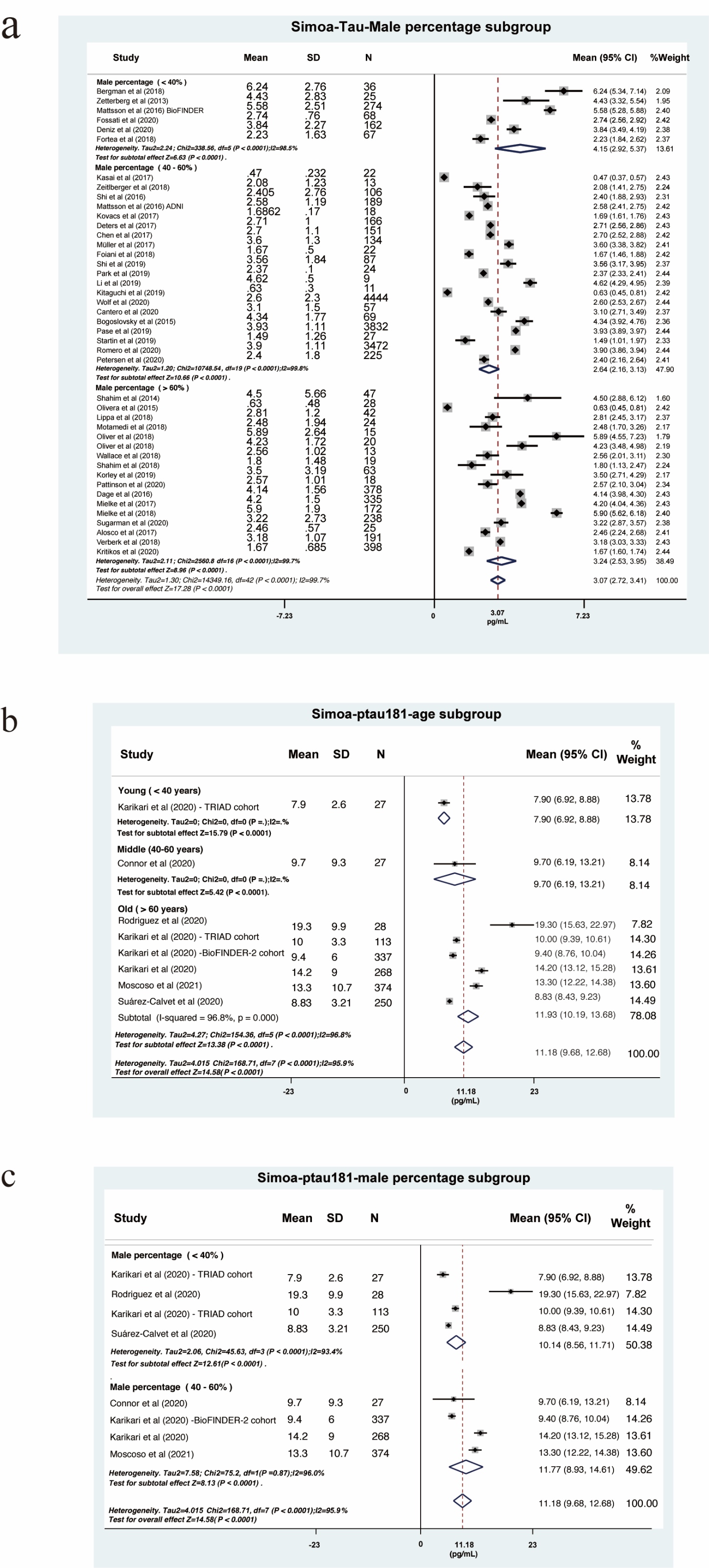


## Figure S2. The levels of plasma tau and ptau181 in healthy people using Simoa in different subgroups. Meta-analysis of studies calculating plasma tau levels of different male percentages (a), and plasma ptau181 levels of different ages (b) and male percentages (c) in the healthy population using Simoa.

**Figure S3.** The level of plasma tau in healthy people using IMR in different subgroups. Meta-analysis of studies calculating plasma tau levels of different age**s** (a) and male percentage**s** (b) in the healthy population using IMR.

## Figure S4. The levels of plasma tau and ptau181 in healthy people using EIMAF/a-EIMAF and MSD. Meta-analysis of studies calculating plasma tau levels of different ages (a) using EIMAF/a-EIMAF and plasma ptau181 levels using MSD (b) in the healthy population.

**Figure S5.** Comparison of plasma tau and ptau181 between AD and healthy controls using Simoa, IMR, and MSD. Meta-analysis of studies comparing plasma tau levels between AD and healthy controls using Simoa by male percentage **(a)** and using IMR **(b)**, and plasma ptau181 levels using MSD **(c)**. AD, Alzheimer’s disease; SD, standard deviation; CI, Conﬁdence Interval; WMD, Weighted Mean Difference.

**Figure S6.** Funnel plot of the random-effect analysis. Funnel plot of plasma tau using Simoa **(a)** and IMR **(b)**, and ptau181 using Simoa **(c)** and MSD **(d)** in AD patients *vs* control.

**Figure S7.** The diagnostic accuracy of plasma ptau181 using MSD. The forest plots of pooled sensitivity and specificity **(a)**, HSROC curve **(b)** and Fagan’s nomogram **(c)** to estimate the clinical utility of ptau181 level using MSD.
